# Editorial: Precision nutrition for lifestyle, health and disease

**DOI:** 10.3389/fmolb.2026.1867849

**Published:** 2026-05-11

**Authors:** Omar Ramos-Lopez, Mayank Garg, Naveen Kumar Bhatraju, Kanika Agarwal, Geeta Trilok-Kumar

**Affiliations:** 1 Medicine and Psychology School, Autonomous University of Baja California, Tijuana, Mexico; 2 Koita Centre for Digital Health (KCDH-A), Ashoka University, Sonipat, Haryana, India

**Keywords:** artificial intelligence, bioinformatics, biomarkers, chronic disease, digital health, omics, precision medicine, precision nutrition

Genetic, environmental and lifestyle factors dynamically interact and shape individual health over time. Modelling these interactions and their contribution to the maintenance of health, disease development and progression is critical to understanding the inter-individual heterogeneity in the health trajectories, and to developing tailored prevention and therapeutic strategies suited to individual health needs. Precision medicine (PM) is an interdisciplinary field that integrates genetic, molecular, environmental, and lifestyle data to inform targeted prevention and therapeutic strategies. Precision nutrition is an emerging sub-domain of PM that deals with how heterogeneous physiological responses to diet contribute to health trajectories. Apart from direct modulation of various metabolic processes, emerging evidence in immuno-metabolism also suggests the role of diet in immune regulation, inflammation, and redox signaling, making it critical for healthcare (Livingstone et al., 2022). Realising this transformative potential requires studies that dissect and characterise dietary responses through these biochemical and immunological pathways. Such an integrative approach could allow discovery of novel biomarkers for risk stratification, early diagnosis, prognosis and designing personalized therapeutics. Recent advances in multiomics, artificial intelligence and machine learning offer an opportunity to integrate relevant high dimensional multimodal data accelerating the translational potential of precision nutrition (Mani et al., 2025). To further this vision, we hosted a Research Topic titled “*Precision Nutrition for Lifestyle, Health and Disease*” to disseminate cutting edge research in the field of precision nutrition, from a mechanistic perspective to its intended application. The broader aim is promoting health and improving the prevention, prediction, management and prognosis of chronic diseases with an integrative and translational approach.

Chronic disorders are gradually becoming the leading cause of mortality and disability worldwide (Di Cesare et al., 2024). While tertiary care is warranted, lifestyle modifications remain the cornerstone for prevention and management of chronic disorders such as cardiovascular diseases. This includes adopting heart-healthy diets, engaging in regular physical exercise, managing stress, and smoking/alcohol cessation. However, recent advances have highlighted the use of nutraceuticals as a complementary strategy to promote cardiovascular health (Ghodeshwar et al., 2023). Moreover, digital health platforms are being increasingly used to deliver and monitor lifestyle change. Two complementary contributions in this Research Topic illustrate both ends of this continuum. McCarthy et al. studied healthcare workers, an understudied population at high cardiometabolic risk. Through a 1-year prospective observational study during the COVID-19 pandemic that enrolled 104 participants, the authors used digital health platforms to capture lifestyle behaviors and gene expression profiles. They found that the study population exhibited similar alterations in lifestyle behaviors as the general community, including a decline in physical activity and persistent subclinical insomnia, which was partially due to the pandemic. Also, the expression levels of 13 genes, including those categorized as nutrient-responsive and related to immune health, had a moderate correlation with lifestyle features including steps, sleep, distance, and insomnia, although the statistical evidence remains weak. This could be attributed to the seasonal and pandemic associated noise within this cohort, which would benefit from larger sample sizes. The study’s methodological contribution is to demonstrate that wearable-anchored precision-health interventions are feasible in healthcare workers, but sustained engagement will require behavioural-design strategies. Wu and Fang complement this clinical-translational perspective with their review of the current evidence on nutritional supplements in cardiovascular protection including omega-3 fatty acids, coenzyme Q10, magnesium, and selenium, evaluating mechanisms of action and clinical applications. They report that these compounds may modulate key pathophysiological processes in cardiovascular disease, such as dyslipidemia, vascular inflammation, oxidative stress, and endothelial dysfunction. However, inconclusive data and inconsistent study designs (dosage, time of intervention, population characteristics) warrant further investigation. Moreover, because universal high-dose regimens are unlikely to be beneficial and may be detrimental in some population groups, the authors argue for a structural shift in how the field is positioned. This requires a precision approach comprising mechanism-informed practice, rational formulation choice, biological response validation, and consistent endpoint monitoring to guide the clinical use of cardiovascular supplements according to specific phenotypes.

As discussed above, such precision approaches require multi-omics integration for a better understanding of the molecular processes underlying the development of diseases and the therapeutic potential of dietary compounds to inform precision nutrition strategies (Mahdavi, 2025). In this regard, Sima et al. explored the molecular mechanisms of resveratrol in treating diabetic foot ulcers (DFU) by using bioinformatics analyses, machine learning algorithms, gene expression assays and molecular docking techniques. They identified cytidine deaminase (*CDA*) and ornithine decarboxylase 1 (*ODC1*) as differentially expressed genes modulating the therapeutic effect of resveratrol on DFU. Resveratrol treatment also mediated alterations in the pathological microenvironment of DFU and consequently influenced its progression. The authors conclude that this research delves deeper into the molecular processes underlying resveratrol therapy for DFU and also provides novel targets for DFU care. On a similar note, to explore precision nutrition in inflammatory conditions, Hou et al. investigated the protective effect and underlying mechanism of garlic oil (GO) on lipopolysaccharide (LPS)-induced acute lung injury (ALI) in mice by developing a nanotechnology-based formulation. The results revealed that GO-nanodisks effectively enabled stable encapsulation of GO, enhanced its bioavailability, and improved its therapeutic efficacy against LPS-induced ALI. The GO-nanodisk treatment reduced lung tissue pathology, lowered inflammatory factor levels, and improved oxidative stress status via Keap1/Nrf2 signaling pathway activation and NF-kappa B pathway inhibition. The authors conclude GO-nanodisk formulation provides a promising theoretical foundation for the encapsulation of oil-based pharmaceuticals against ALI.

The operational backbone for translating precision nutrition to clinical practice requires identification of key biomarkers. Biomarkers are measurable biological indicators derived from molecular, histologic, radiographic, or physiologic characteristics that can be used to characterise normal biological processes, pathogenic progressions, or responses to particular exposures or interventions (Califf, 2018). In this Research Topic, Li et al. aimed to identify biomarkers and mechanisms associated with arachidonic acid metabolism (AAM) in vitiligo using bioinformatics tools. In training and validation sets, the AAM-related genes (*PTGDS*, *PNPLA8*, and *MGLL)* were differentially expressed between the lesional and control groups, which showed potential for predicting the risk of vitiligo. These findings suggest that AAM may be closely related to vitiligo pathogenesis and that the AAM-related genes can be used not only as diagnostic biomarkers of this disease, but also as promising therapeutic targets. Furthermore, Zhou et al. explored the prognostic value of inflammation-nutrition biomarkers in patients with lung cancer and tuberculosis. The final model included diabetes, Eastern Cooperative Oncology Group-Performance Status (ECOG-PS), neutrophil-to-lymphocyte ratio (NLR), prognostic nutritional index (PNI), hemoglobin-to-red blood cell distribution width ratio (HRR) and red cell distribution width (RDW) as independent prognostic biomarkers. The authors conclude that this model provided a quantitative tool to stratify individual disease risk and offered evidence for the usage of nutrition-based interventions in susceptible patients.

In summary, the articles in this Research Topic span the breadth of contemporary precision nutrition research. Collectively, they demonstrate the value of combining precision health and lifestyle/nutrition approaches to deliver personalized recommendations for the control and management of chronic diseases. They also delve into the pathophysiological and molecular mechanisms underlying the onset and development of some chronic diseases through the identification of therapeutic targets. Furthermore, they present evidence of the potential of using biomarkers to improve disease prediction and clinical prognosis. The contributions also identify priorities for the field going forward, including larger, adequately powered cohorts and study designs that support causal inference, particularly for nutraceutical and supplementation trials. Additionally, integrating multimodal biological information through artificial intelligence and bioinformatics tools will be essential to translating this knowledge into practical applications of precision nutrition for enhancing health status and improving the holistic management of diseases.
